# Garlic Exosomes Promote Hair Growth Through the Wnt/β-catenin Pathway and Growth Factors

**DOI:** 10.7759/cureus.42142

**Published:** 2023-07-19

**Authors:** Esma Inan Yuksel, Demet Cicek, Betul Demir, Kazim Sahin, Mehmet Tuzcu, Cemal Orhan, Ibrahim Hanifi Ozercan, Fikrettin Sahin, Pelin Kocak, Merve Yildirim

**Affiliations:** 1 Department of Dermatology, Biruni University Hospital, Istanbul, TUR; 2 Department of Dermatology, Faculty of Medicine, Firat University, Elazig, TUR; 3 Department of Animal Nutrition, Faculty of Veterinary Medicine, Firat University, Elazig, TUR; 4 Department of Biology, Faculty of Science, Firat University, Elazig, TUR; 5 Department of Pathology, Faculty of Medicine, Firat University, Elazig, TUR; 6 Department of Genetics and Bioengineering, Yeditepe University, Istanbul, TUR; 7 Department of Biomedical Engineering, Faculty of Engineering and Natural Sciences, Istinye University, Istanbul, TUR

**Keywords:** wnt signaling pathway, vascular endothelial growth factor, transforming growth factor beta, hair follicle, garlic, exosome

## Abstract

Background

Exosomes are membrane-derived nanovesicles produced by cells and play an important role in intercellular communication.

Objectives

This study aimed to investigate the effects of garlic exosome (GE) on hair growth.

Methods

Forty-two Sprague-Dawley/Wistar albino rats were randomly divided into six groups: non-shaved control, shaved control, topical control, GE 2 mg, GE 4 mg, and topical GE. At the end of the experiment, the number of hair follicles, follicle diameter, and subcutaneous tissue thicknesses were measured histopathologically. The Wnt-1, β-catenin, platelet-derived growth factor (PDGF), vascular endothelial growth factor (VEGF), transforming growth factor-β1 (TGF-β1), and collagen I levels were measured by the Western Blot method.

Results

The anagen follicle counts of the GE 2 mg, 4 mg, and topical GE groups were 66.57±15.49, 105.71±25.06, and 55.29±6.72, and were significantly higher than the control groups (p<0.01, p<0.001 and p<0.05, respectively). The follicle diameter of the GE 4 mg group was higher than the others (p<0.05). The Wnt-1, PDGF, VEGF, TGF-β1, and collagen I levels of all GE groups, and the β-catenin levels of the GE 4 mg and topical GE groups were significantly higher than the control groups (p<0.05).

Conclusion

GE induces hair growth in rats via the Wnt-1, β-catenin, VEGF, PDGF, and TGF-β1 signaling pathways.

## Introduction

The process of hair growth involves distinct cycles, including anagen, catagen, and telogen phases. These cycles result from complex communications between the epidermal placode, which is composed of keratinocytes and the dermal papilla, which originates from mesenchyme. Numerous signalling pathways and growth factors are involved in this communication. The activation of the Wnt/β-catenin signalling pathway significantly contributes to the initiation of the anagen phase in humans. While many growth factors, including insulin-like growth factor-1, platelet-derived growth factor (PDGF), keratinocyte growth factor, hepatocyte growth factor, vascular endothelial growth factor (VEGF), fibroblast growth factor-7 (FGF-7), epidermal growth factor are involved in the maintenance of the anagen phase, the transition to the catagen phase is primarily regulated by FGF-5 and transforming growth factor-β (TGF-β) [1].

Hair loss is an important dermatological disease that damages self-esteem in both men and women and reduces personal attractiveness and therefore can cause some problems in social communication and psychological well-being. Many conditions such as certain physiological factors, endocrine and metabolic disorders, nutritional disorders, trauma, drugs, and autoimmune diseases can cause telogen and anagen effluvium. Currently, the US Food and Drug Administration (FDA) has granted approval for two drugs, finasteride and minoxidil, for the treatment of hair loss in men. However, the cure rates of these drugs are not satisfactory, and their therapeutic use is limited due to some local and systemic side effects [2].

Garlic (*Allium sativum* L. fam. *Alliaceae*) is a bulbous plant that has been widely used to treat many health problems for centuries and is currently the subject of extensive research. It is known to have antioxidant, immunomodulatory, anticancer, antiatherosclerotic, antihypertensive, and antidiabetic effects. Today, garlic is added to hair care products as a topical and oral supportive agent and is widely used in treating alopecia areata in traditional medicine worldwide [3].

Exosomes are small membrane-derived extracellular nanovesicles ranging in size from 30 to 100 nm produced and released by cells [4]. Many cells of animal and plant origin can secrete exosomes. Exosomes contain different proteins, mRNA, and micro (mi) RNAs depending on the cell from which they originate, and they are crucial for intercellular communication [4]. While studies have demonstrated that exosomes derived from human dermal papilla cells can induce the transition to anagen from telogen, and also prolong the anagen phase, there is currently no research indicating the impact of plant-derived exosomes on hair growth [5]. Plant-derived exosomes have demonstrated utility in various areas, including cancer therapy, modulating the intestinal microbiota, and treating inflammatory bowel diseases [6, 7]. Recent studies have shown that garlic exosomes (GEs) can be easily absorbed from liver cells and inhibit cancer cell proliferation by inducing apoptosis [6, 8].

The objective of this study was to investigate the effects of oral and topical GEs on hair growth in rats and examine their impacts on the Wnt-1/ β-catenin, PDGF, VEGF, and TGF-β1 signalling pathways involved in the hair cycle.

## Materials and methods

Experimental animals and study design

Forty-two Sprague-Dawley/Wistar female albino rats with an age of 8 weeks and a mean weight of 180 ± 20 g were obtained from Firat University Experimental Research Center. Throughout the experiment, the rats were maintained under controlled environmental conditions, including a temperature of 23 ± 2°C, humidity of 55 ± 10%, and a 12/12-hour light-dark cycle with automatic illumination. The rats were provided with food and water ad libitum. The study was conducted in accordance with the established ethical guidelines for the use and care of laboratory animals, as outlined in the European Economic Community guidelines [9].

The rats underwent a one-week acclimation period prior to the commencement of the study. Considering the possible effects of shaving and the topical carrier agent on hair follicles and skin, the rats were randomly assigned to six groups in order to ensure unbiased distribution for the study. These groups consisted of three control groups: non-shaved, shaved, and topical control (shaved + vehicle), as well as three study groups: GE 2 mg (shaved + GE 2 mg/kg/day), GE 4 mg (shaved + GE 4 mg/kg/day), and topical GE (shaved + topical GE 4 g + vehicle). In the topical form, 0.5% carboxymethyl cellulose + 95.5 g distilled water were used as carriers. The specified doses of exosomes were applied daily through the peroral route using the gavage method in the GE 2 mg and GE 4 mg groups, while the spray form was topically administered to the topical GE group. Following the completion of the 42-day period, euthanasia was performed on all rats through cervical dislocation, dorsal skin samples were collected, and then histopathological examination and molecular analysis were performed.

Exosome isolation and characterization

Garlic juice was used to isolate nanovesicles. *A. sativum* was obtained from Taşköprü, Kastamonu, Turkey, and rinsed in distilled water before being pressed in a laboratory blender. For 10 minutes, the garlic juice was centrifuged at 300 g. The supernatant was transferred to fresh tubes and subjected to spinning at 16,000 g for a period of 30 minutes. The Exo-spin™ buffer (Cell Guidance Systems, California, USA) was added to the supernatant at half the volume, followed by incubation at a temperature of 4 °C for a period of 2 hours. Then, the samples underwent centrifugation for one hour at 16,000 g. The pellet was filtered using a 0.22-m pore filter and suspended in phosphate buffer (PBS) (Exo-spin™ Exosome Purification Kit, UK). Using the Lowry assay, nanovesicle quantification was performed (Bio-Rad, USA). Then, nanoparticle tracking analysis (NTA), Zetasizer (Malvern Instruments Ltd., Malvern, Worcestershire, UK), flow cytometry, and scanning electron microscopy (SEM) were performed for the examination of the isolated exosomes.

Size distribution analysis of exosomes with NTA and Zetasizer

Nanosight and Zetasizer were used to determine the size distribution and particle count of the exosome-like nanoparticles (Malvern Instruments Ltd., Malvern, Worcestershire, UK). These parameters were examined at room temperature following the dilution of the samples in 1 ml of distilled water. Exosome-like nanoparticles were exposed to a blue laser beam at a wavelength of 488 nm, and a video was captured at a frame rate of 25 frames per second. NanoSight NTA software (Malvern Instruments), version 3.3, was used to examine the footage. Each exosome-like nanoparticle replication was examined at least five times, and the two-dimensional Stokes-Einstein equation was utilized to determine the particle size of exosomes based on both the Brownian motion and the particle movement velocity. The particle size of the isolated exosomes was further assessed using Zetasizer Nano ZS (Malvern Instruments).

Exosome characterization with scanning electron microscopy

The first step was to vortex and resuspend freshly separated exosomes in 0.2-1 ml of Dulbecco's PBS. The samples were fixed in an aqueous 2% paraformaldehyde solution of EMS (electron microscopy grade) quality and introduced into cleaned silicon chips that had been cleansed with water, dried in vesicles beneath a ventilation hood, and immobilized after a series of dilutions with distilled water. The samples were then attached to the prepared silicon chips on a SEM stage applying carbon paste. Before SEM imaging, a 2-5-nanometer gold-palladium alloy coating was sputtered over the surface to make it conductive. For SEM imaging, low beam energies ranging from 5 to 10 kV were employed. R/Bioconductor was used to estimate the density distribution of exosome diameters, and ImageJ(National Institute of Health, Bethesda, USA) was used to examine the sizes of the SEM pictures.

Histopathological examination

At the end of the experiment, dorsal skin samples (2 x 2 cm) were separated rapidly and fixed in 10% formalin. After routine processing and embedding of the dorsal skin samples in paraffin blocks, sections with a 4-µm thickness were prepared. Then, the sections underwent the hematoxylin and eosin (H&E) staining process and were examined by a blinded pathologist using a light microscope (Olympus BX53, Olympus Corporation, Tokyo, Japan). Using a 10x objective, the number of follicles was determined in a 7-mm² area. Measurements were taken for the mean diameter of anagen follicles, as well as the thicknesses of the epidermis, dermis, and subcutaneous tissue. The Olympus cellSens Standard system (version 1.16; Olympus Corporation, Tokyo, Japan) was used to perform digital photomicrography and measurements.

Molecular analysis

The Western Blot technique was employed to measure the levels of collagen I, Wnt-1, β-catenin, VEGF, PDGF, and TGF-β1 from the dorsal skin, in order to determine the effect of GE on the pathways associated with hair growth. The dorsal skin samples were homogenized in an extraction buffer at 4 °C, then subjected to centrifugation at 13,000 x g for 20 minutes at 4 °C. After the separation of proteins using a 10% SDS-PAGE (sodium dodecyl sulfate-polyacrylamide gel electrophoresis) gel, they transferred to cellulose nitrate membranes for one hour. The cellulose nitrate membranes were rinsed with PBS twice each for 5 minutes. Prior to administration, the primary antibody was blocked at room temperature for one hour using PBS, which contains 1% bovine serum albumin. Afterwards, specific antibodies against collagen I, Wnt-1, β-catenin, PDGF, VEGF, and TGF-β1 (Santa Cruz Biotechnology, California, USA) were used to incubate membranes overnight at 4 °C. After washing the membranes with 0.1% Tween 20 in Tris-buffered saline, they were subjected to a 1-hour incubation with secondary goat anti-mouse antibodies (Santa Cruz Biotechnology). The antibody β-actin (Abcam, Cambridge, UK) was used to control protein loading control. For the densitometric analysis of the bands, the ImageJ image analysis system was used.

Statistical analysis

The statistical analysis of the data was performed using the Statistical Package for the Social Sciences (SPSS, version 25.0. Armonk, NY: IBM Corp.). Mean and standard deviation values were used in the expression of the numerical data. One-way analysis of variance with post hoc Tukey test was used for multiple comparisons, and a level of p<0.05 was used to determine statistical significance.

## Results

Exosome detection and visualization with scanning electron microscopy

**Figure 1:**
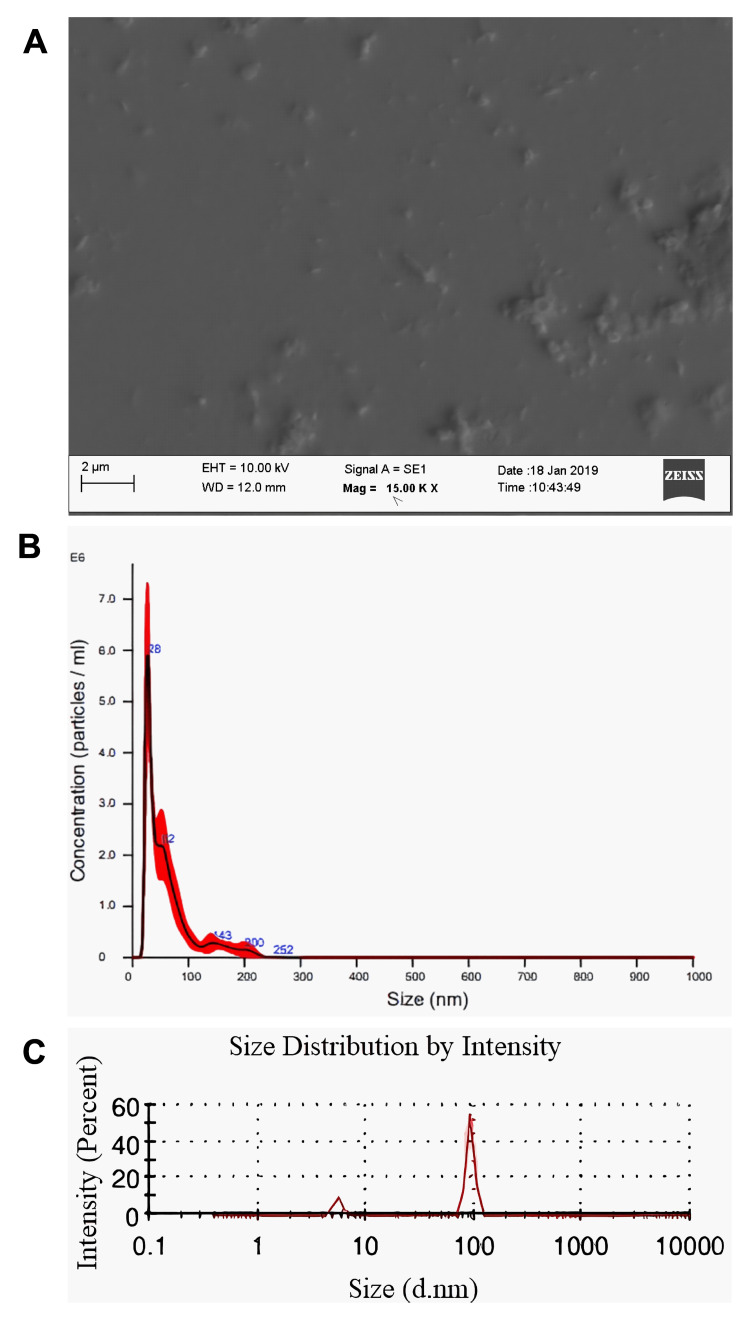
Exosome detection and visualization with scanning electron microscopy. (A) Scanning electron microscope of garlic-derived nanovesicles isolated with the Exo-spin™ Exosome Purification Kit (UK) for characterization. (B) Nanoparticle tracking analysis of garlic-derived nanovesicles isolated with the Exo-spin™ Exosome Purification Kit (UK) for characterization. (C) Zetasizer analysis of garlic-derived nanovesicles isolated with the Exo-spin™ Exosome Purification Kit (UK) for characterization (d.nm: diameter nanometer).

The size of the isolated vesicles ranged from 30 nm to 100 nm in the SEM analysis (Figure 1A, 1C). Confirmation was also made using NTA and Zetasizer, which showed that the garlic-derived nanovesicles varied in size between 30 and 100 nm (Figure 1B). Hence, the findings demonstrated that the nanovesicles in garlic juice exhibited exosome-like characteristics in terms of size and morphology.

Histopathological examination

**Figure 2:**
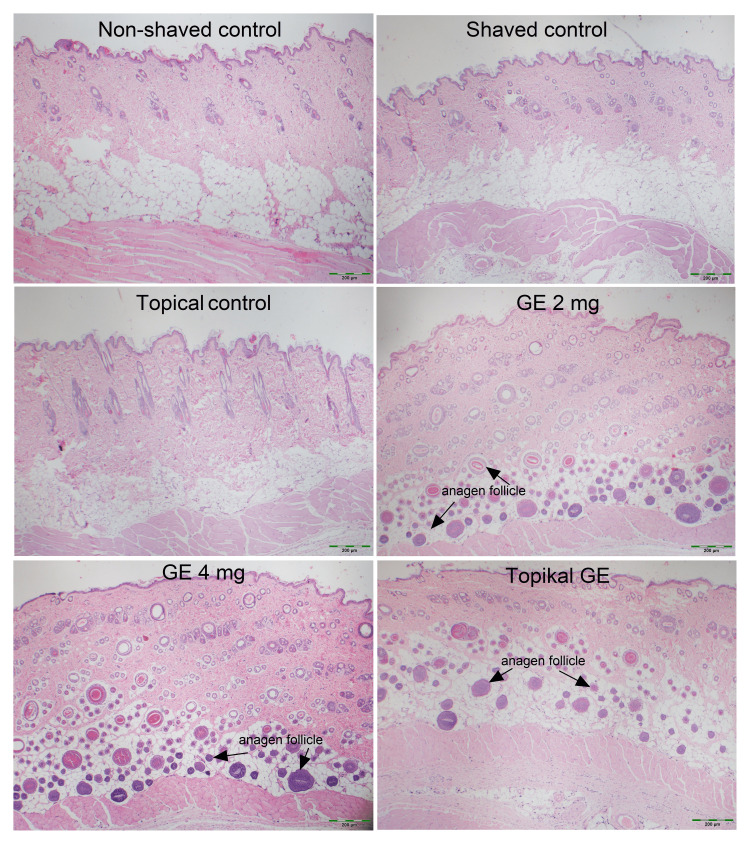
Effect of GE (garlic exosome) on anagen follicle count, telogen follicle count, follicle diameter, and epidermis, dermis, and subcutaneous tissue thicknesses in the histological examination of dorsal skin in rats (H&E, 40x).

The numbers of anagen follicles in study groups were calculated as 66.57 ± 15.49 in GE 2 mg, 105.71 ± 25.06 in GE 4 mg, and 55.29 ± 6.72 in topical GE. In comparison to the numbers obtained from control groups, the anagen follicle counts were significantly higher in all study groups (p < 0.01, p < 0.001, and p < 0.05, respectively). In the histopathological examination, it was observed that none of the control groups exhibited anagen follicles in the subcutaneous tissue (Table 1, Figure 2).

**Table 1:** Effects of GE on the skin in rats according to the histological examination GE: Garlic exosome. Data are presented as mean and standard error. Different superscript letters indicate groups that have statistically different mean values, while the same superscript letters indicate groups that are statistically similar (a-c) (p < 0.05). ‡Follicle diameter was not measured in this group because there were no anagen follicles in these rats.

Histopathological findings	Groups					
	Non-shaved control	Shaved control	Topical control	GE 2 mg	GE 4 mg	Topical GE
Follicle count (1/1 mm^2^)	41.00 ± 7.15^a^	57.42 ± 10.89^a^	83.43 ± 6.69^ab^	144.28 ± 31.97^bc^	170.71 ± 29.93^c ^	105.14 ± 13.65^abc^
Anagen (1/1 mm^2^)	0.00 ± 0.00^a^	0.00 ± 0.00^a^	0.00 ± 0.00^a^	66.57 ±15.49^b^	105.71 ± 25.06^b^	55.29 ±6.72^b^
Telogen (1/1 mm^2^)	41.00 ± 7.15^a^	57.42 ± 10.89^a^	83.43 ± 6.69^a^	77.71 ± 18.94^a^	79.29 ± 13.18^a^	49.86 ± 7.78^a^
Follicle diameter (µm)	-^‡^	-^‡^	-^‡^	31.42 ± 3.29^a^	59.65 ± 4.96^b^	45.70 ± 2.38^c^
Epidermis (µm)	11.05 ± 1.12^a^	11.87 ± 1.32^a^	12.71 ± 0.70^a^	11.71 ± 0.74^a^	11.51 ± 1.25^a^	13.11 ± 1.16^a^
Dermis (µm)	616.00 ± 64.69^a^	605.28 ± 56.67^a^	725.85 ± 57.73^a^	608.28 ± 45.34^a^	651.28 ± 22.29^a^	605.14 ± 28.43^a^
Subcutaneous tissue (µm)	253.42 ± 33.50^ab^	178.42 ± 19.98^b^	215.42± 36.01^bc^	342.00 ± 31.79^ac^	314.42 ± 28.47^ac^	371.14 ± 31.01^a^

The follicle diameters of the GE 2 mg, GE 4 mg, and topical GE groups were 31.42 ± 3.29, 59.65 ± 4.96, and 45.70 ± 2.38 µm, respectively. The follicle diameter is higher in the group of GE 4 mg compared to the GE 2 mg and topical GE groups; the difference was statistically significant (p < 0.001 and p < 0.01, respectively). Significantly higher follicle diameter was found in the group with topical GE compared to the GE 2 mg (p < 0.01). No significant difference was detected among the groups in terms of telogen follicle count (p > 0.05) (Table 1, Figure 2).

Molecular analysis

**Figure 3:**
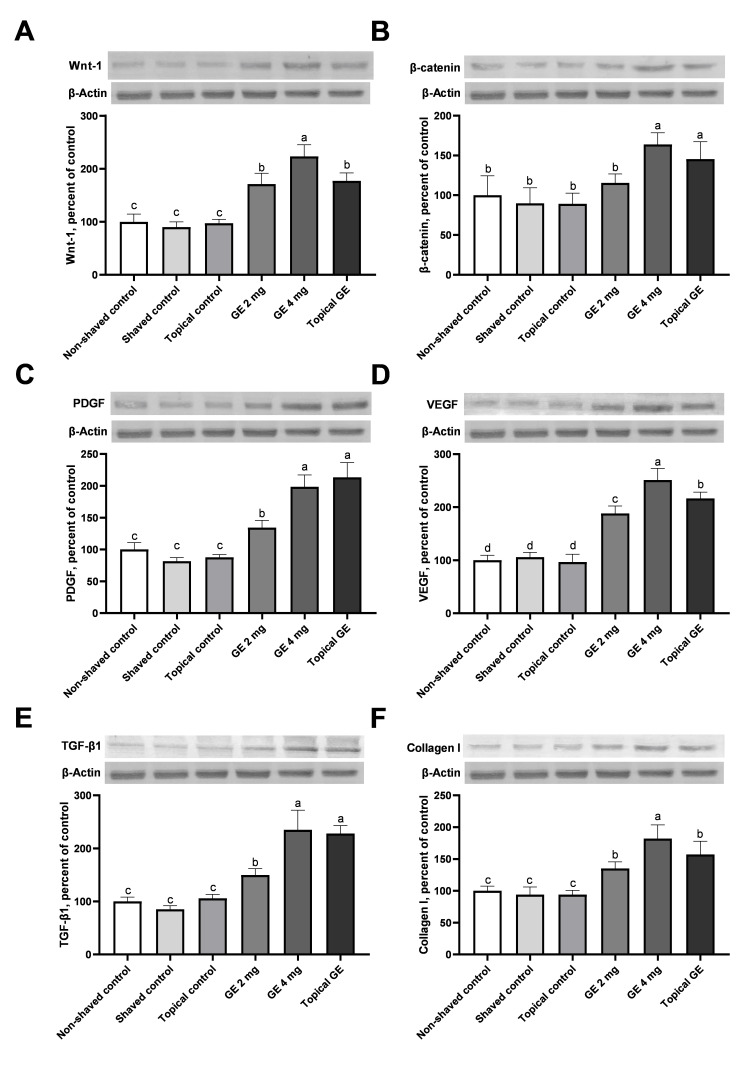
Effects of garlic exosome on the Wnt-1 (Panel A), β-catenin (Panel B), PDGF (Panel C), VEGF (Panel D), TGF-β1 (Panel E), and collagen I (Panel F) protein levels in the dorsal skin of rats. Signal intensities in Western Blots were evaluated with the densitometric analysis. Data are expressed as the ratio of the treatment value to the control value. Western Blot measurements were repeated at least three times. β-actin was used as a control. The bars represent mean and standard error values. Differences in the mean values between the groups are indicated by different superscripts (a-c) (p < 0.05). PDGF: platelet-derived growth factor; VEGF: vascular endothelial growth factor; TGF-β1: transforming growth factor-β1.

The Wnt-1, β-catenin, VEGF, PDGF, TGF-β1, and collagen I, levels measured from the dorsal skin are presented in Figure 3. The Wnt-1, VEGF, PDGF, TGF-β1, and collagen I protein levels were significantly higher in the GE 2 mg group than in the control groups. The Wnt-1, β-catenin, VEGF, PDGF, TGF-β1, and collagen I protein levels were significantly higher in the GE 4 mg and topical GE groups than in the control groups. Similar levels of the β-catenin, TGF-β1, and PDGF were noted between the topical GE and GE 4 mg group, but they were significantly higher compared to the GE 2 mg group (Figure 3).

## Discussion

It has been shown that exosomes easily transfer their contents, such as miRNA, protein, and active lipids, and they regulate gene and protein expression in recipient cells. Recently, exosomes have been obtained from various plants, such as garlic, grapefruit, ginger, lemon, tomato, and apple, and these plant-derived exosomes have been reported to easily transfer their contents to animal cells [10]. Human dermal papilla cell-derived exosomes, dermal exosomes containing miRNAs, and adipose-derived stem cell exosomes have been shown to induce anagen from telogen and also prolong the anagen phase [5, 11, 12]. However, there is currently no study showing the specific effect of plant-derived exosomes on hair growth. Considering this information, we conducted the current study using GEs in peroral and topical formulations in rats to investigate the potential effect of GE on hair growth.

This study determined that GEs resulted in significant anagen induction in rats and activated signaling pathways implicated in hair growth. Peroral GE increased the number of anagen follicles and the expression of Wnt-1, VEGF, PDGF, and TGF-β1 in the rats even at a low dose (2 mg/kg/day). In the rats receiving a high dose of peroral GE (4 mg/kg/day), the follicle diameter was higher, and the activation of signaling pathways was more pronounced. The topical GE application not only increased anagen follicle growth equivalent to the low-dose peroral GE but also increased the follicle diameter more than the low-dose peroral GE, and stimulated the β-catenin, VEGF, PDGF, and TGF-β1 pathways to a greater extent. Our findings demonstrate that topical GE can be absorbed through the skin, and even its biological effects on the skin are similar to its peroral use.

Although garlic is widely used in the treatment of alopecia in traditional medicine across the world there is not yet sufficient scientific evidence concerning the positive effect of garlic on hair growth. Maluki et al. and Hajheydari et al. applied a topical garlic extract to alopecia areata and demonstrated the induction of hair growth [13, 14]. Recently, Fares et al. showed that topical garlic, whose absorption was increased with the phonophoresis method, provided more epidermal regeneration and further increased mesenchymal stem cells, and there was a significant increase in hair follicles in these rats [15]. It is not yet known by which mechanisms garlic stimulates hair growth. Garlic contains many important components, some of which are also involved in hair growth. Allicin, an organosulfur, is considered responsible for many of the biological effects of garlic. Garlic also contains sulfur-containing compounds, such as DADS (diallyl disulfide), DATS (diallyl trisulfide), and alliin; various enzymes such as alliinase; vitamins A, C, and B-complex; amino acids, such as histidine, arginine, cysteine, methionine, and phenylalanine; flavonoids; and high amounts of potassium, zinc, sulfur, and selenium [16]. The therapeutic effect of garlic in alopecia areata is related to its anti-inflammatory, immunomodulatory, and antioxidant activities. Song et al. showed that GE exerted an anti-inflammatory effect on Hep G2 cells by reducing interferon gamma and interleukin-6 levels in vitro [6]. Garlic has been shown to inhibit the 5α-reductase enzyme potently, and this antiandrogenic activity may explain its possible curative on androgenetic alopecia [17].

The significant involvement of the Wnt/β-catenin signaling pathway in hair follicle morphogenesis and regeneration is well known. Wnt-3a, 5a and 10b are proteins with a well-known role in hair growth [18, 19]. During embryogenesis and tissue differentiation, Wnt-1 exhibits important functions. The overexpression of Wnt-1 has been linked to tumorigenesis, but its specific involvement in hair growth still requires further investigation [20]. In the present study, we provided evidence that utilization of both oral and topical formulations of GE resulted in the activation of the Wnt-1/β-catenin signaling pathway leading to the induction of the anagen phase in rats. The anti-inflammatory properties of DADS, DATS, and S-allyl cysteine in garlic have been demonstrated through their ability to suppress the NF-κB (nuclear factor kappa) and MAPK (mitogen-activated protein kinase) signaling pathways, leading to a reduction in lipopolysaccharide-induced inflammatory mediators. [21]. NF-κB also has a complex relationship with the Wnt family and is involved in developing and limiting epidermal placode formation [22]. Garlic is likely to exert its placode formation-inducing effect over Wnt through the inhibition of NF-κB.

In the dermal papillae and outer root sheath, VEGF maintains the anagen cycle through its ability to stimulate fibroblast proliferation and dermal microcirculation [23]. Our findings revealed that the administration of GE led to an upregulation of VEGF expression, which can be attributed to its effect on inducing the anagen phase. In parallel to this, the VEGF levels increased in the rats with a high follicle diameter. These findings agree with previous studies showing that VEGF is crucial in determining the diameter of follicles, and therefore hair thickness [23, 24].

TGF-β is a regulatory protein family involved in cell proliferation and differentiation and may show different functions in different tissues In human hair follicles, the presence of TGF-β1 and 2 triggers the transition from the anagen phase to the catagen, with TGF-β1 playing a significant role in the development of androgenetic alopecia [25]. The impact of TGF-β1 on hair growth in rats is not known. We observed that the administration of GE in rats resulted in the activation of TGF-β1 while inducing hair growth.

We found that oral and topical GEs increased collagen I levels in rats. It has been shown that garlic accelerates wound healing while also increasing fibroblast proliferation and the maturation of collagen bands, and accelerating reepithelialization [26]. Dimethyl sulfone and many other garlic ingredients can increase collagen synthesis by providing a source of sulfur, which is vital for synthesis. In addition to increasing collagen synthesis, garlic has been shown to reduce collagen degradation by reducing free oxygen radical production and matrix metalloproteinase-1 (MMP-1) in keratinocytes exposed to ultraviolet B, thus preventing photoaging [27].

Human-derived exosomes are on the horizon as an exciting therapeutic for alopecia treatment. This study reveals that GE is a promising herbal agent in the treatment of hair loss in humans. The important advantage of using GE in the treatment of hair diseases is that they are cell-free products that do not carry the risk of microbial contamination. Exosomes are also suitable for commercial use because they can be stored at low temperatures and easily transported to patients [28]. The oral use of garlic products may cause anaphylaxis, angioedema, urticaria, pemphigus, allergic contact dermatitis, photoallergy, and may lead to changes in platelet function [29]. Topical garlic extracts can cause allergic contact dermatitis, and when used in under-occlusive dressings, their diallyl disulfide (DADS), allicin, and allyl propyl disulfide contents can result in coagulation necrosis, albeit rarely [30]. It has been shown that plant-derived exosomes can be highly absorbed and cause fewer immunological reactions [28]. Similarly, we showed that the anagen induction effect of topical GE was similar to its peroral use and we did not observe any skin reaction such as erythema in rats given topical GE. The use of topical GE in treating hair diseases can prevent possible side effects associated with the systemic and topical use of other formulations obtained from garlic extracts.

The notable important limitation of this study is that GE was not compared to systemic and topical garlic extracts in different formulations.

## Conclusions

In conclusion, peroral and topical GEs induce anagen via the signaling pathways of Wnt-1, β-catenin, VEGF, PDGF, and TGF-β1 in rats. The anagen induction effect of topical GE is similar to its peroral use. Further studies on the use of GE in humans will reveal the place of systemic and topical GE use in treating hair diseases.
